# Surgical Treatment of Dysphagia Secondary to Anterior Cervical Osteophytes Due to Diffuse Idiopathic Skeletal Hyperostosis

**DOI:** 10.3390/medicina58070928

**Published:** 2022-07-13

**Authors:** Ho Yong Choi, Dae Jean Jo

**Affiliations:** Department of Neurosurgery, Kyung Hee University Hospital at Gangdong, College of Medicine, Kyung Hee University, Seoul 05278, Korea; apuzzo@hanmail.net

**Keywords:** diffuse idiopathic skeletal hyperostosis, dysphagia, osteophyte, cervical spine

## Abstract

Diffuse idiopathic skeletal hyperostosis (DISH) is an ossifying and ankylosing skeletal condition that can be associated with DISH-related dysphagia in the case of cervical involvement. In contrast to most cases of dysphagia, which are managed conservatively, DISH-related dysphagia can be discouraging due to the progressive nature of DISH. We report two cases of DISH-related dysphagia that were treated with the surgical removal of osteophytes via an anterolateral approach. We were able to remove osteophytes using the bottleneck point as an anatomical landmark between the vertebral body and the bony excrescence. Patients’ symptoms improved following osteophyte removal, without recurrence. In cases of DISH-related dysphagia, osteophyte removal using an osteotome could improve dysphagia safely and quickly.

## 1. Introduction

Diffuse idiopathic skeletal hyperostosis (DISH), also known as Forestier disease, is an ossifying and ankylosing skeletal condition characterized by ossification of ligaments, ossification of tendons, and entheses in various regions of the body [1]. The radiographic characteristics of DISH include soft tissues of the anterolateral thoracolumbar spine ossified over at least four contiguous segments, with flowing vertebral bony bridges without obvious signs of intervertebral or apophyseal degenerative changes. The prevalence of DISH is reported to be as high as 27.1%, and it most commonly involves the anterolateral cervical spine in up to 78% of cases [2,3,4]. The etiology of this condition is unknown; however, strong correlations with metabolic disorders, especially high body mass index and insulin-independent diabetes mellitus, have been documented [5].

Cervical involvement in DISH can be either asymptomatic or associated with various symptoms, including dysphagia, dyspnea, dysphonia, foreign body sensation, hoarseness, stridor, spinal rigidity, cervical pain, and neurological signs caused by medullary compression [1]. Dysphagia is one of the most common symptoms in patients with DISH, and its prevalence has been reported to be 17–25% in patients with DISH affecting the cervical spine [3,6,7,8]. In addition to mechanical compression by osteophyte formation, various mechanisms have been proposed to result in DISH-related dysphagia, including local inflammation, osteophyte-induced muscle spasm, restriction of movement of the epiglottis and larynx, and retention of food in the pyriform sinus due to indentation of the pharynx.

In contrast to most cases of dysphagia, which are managed by conservative measurements, DISH-related dysphagia can be discouraging due to the progressive nature of DISH [9,10]. Although the optimal treatment of DISH-related dysphagia has not been established due to the rarity of this condition, previous studies have reported favorable outcomes following surgical excision of the osteophytes [1,4,11,12].

We report two cases of DISH-related dysphagia that were successfully treated with surgical removal of osteophytes via an anterolateral approach. We were able to remove osteophytes safely and quickly using the bottleneck point as an anatomical landmark between the vertebral body and the bony excrescence.

## 2. Case Reports

### 2.1. Case 1

**Figure 1:**
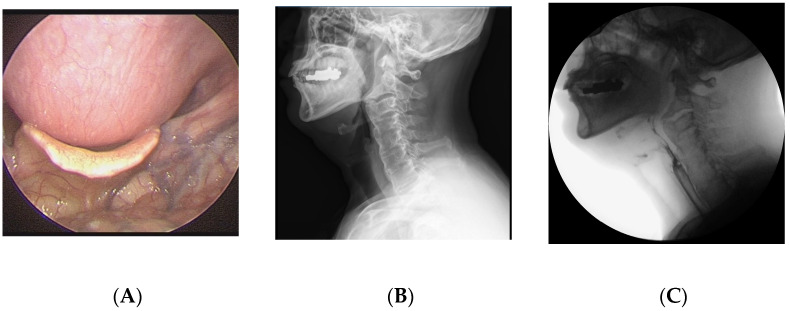

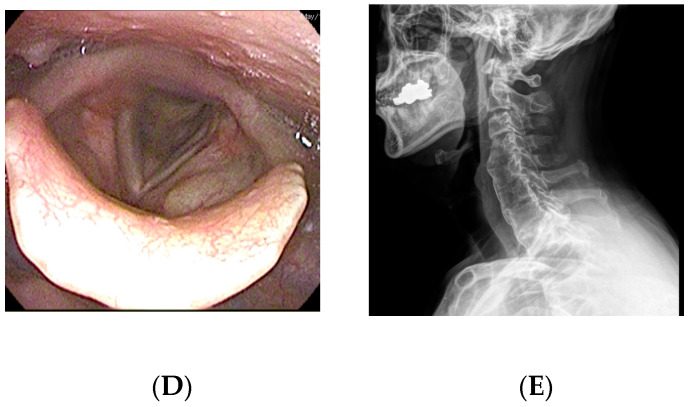
A 64-year-old man presented with swallowing difficulty for the past 2 months. (**A**) Preoperative laryngoscopy revealed a huge protruding mass at the posterior pharyngeal wall. (**B**) Lateral cervical radiograph showed continuous irregular hyperostosis along the anterior aspect of the cervical and upper thoracic vertebral bodies, suggesting diffuse idiopathic skeletal hyperostosis (DISH), with a beak-shaped osteophyte compressing the posterior pharynx. (**C**) Preoperative visual fluoroscopic swallowing study revealed moderate dysphagia in the oral and pharyngeal phases with incomplete closure of the epiglottis. (**D**) Postoperative laryngoscopy showed disappearance of the protruding mass at the posterior pharyngeal wall. (**E**) One-year postoperative radiograph showed no evidence of regrowth of the osteophyte.

A 64-year-old man visited the department of otolaryngology presenting with swallowing difficulty for the past 2 months. Although he was able to have an oral diet, he felt difficulty in swallowing solid food and showed severe aspiration tendency in swallowing liquid food. His medical history included atrial fibrillation and he was on digoxin. On laryngoscopic examination, a large protruding mass was identified in the posterior pharyngeal wall (Figure 1). Lateral cervical radiography and computed tomography (CT) revealed continuous irregular hyperostosis along the anterior aspect of the cervical and upper thoracic vertebral bodies, suggesting DISH with a beak-shaped osteophyte compressing the posterior pharynx. Preoperative visual fluoroscopic swallowing study (VFSS) revealed moderate dysphagia in the oral and pharyngeal phases with incomplete closure of the epiglottis. For solid food, a small amount (<10%) of residue was demonstrated. For liquid, direct aspiration to the trachea was identified. He was referred to the spine center for surgical removal of the osteophyte, which was causing dysphagia. After conventional transverse anterolateral skin incision, the osteophyte was removed via the modified Smith–Robinson approach. The osteophyte was using osteotomes starting from the groove between the vertebral body and the osteophyte. The surgery took 60 min, and blood loss was less than 50 mL. Bone wax was applied to the bony margin after osteophyte removal for hemostasis as well as preventing recurrent osteophytes. His symptoms markedly improved immediately after the surgery. He felt no discomfort swallowing solid or liquid food. Postoperative laryngoscopy showed the disappearance of the mass lesion at the pharyngeal wall and widening of the pharyngeal space. There was no evidence of osteophytes at 1-year postoperative follow-up.

### 2.2. Case 2

**Figure 2:**
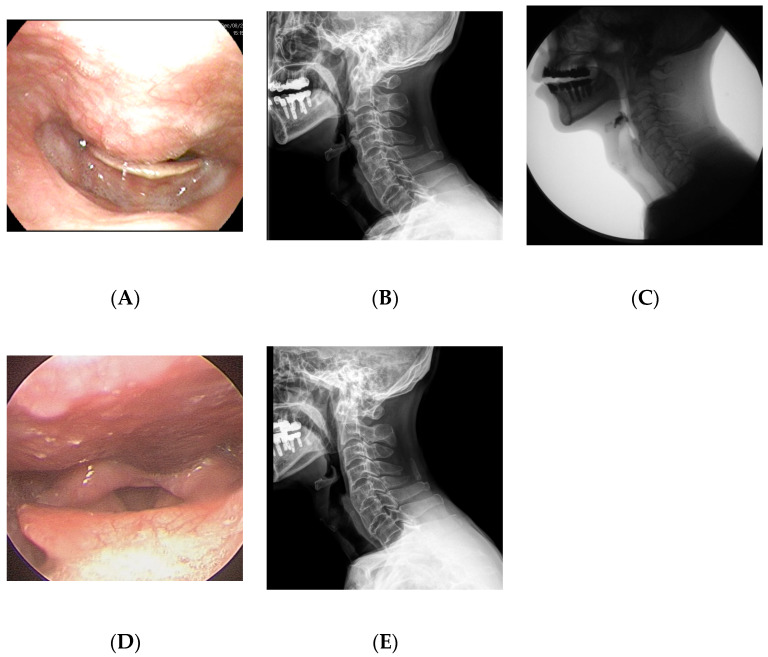
A 65-year-old man presented with dysphagia and odynophagia for the past 3 months and 12 kg weight loss. (**A**) Preoperative laryngoscopy revealed a protruding mass at the posterior pharyngeal wall obstructing the laryngeal entrance. (**B**) Lateral cervical radiograph showed a bridging osteophyte at the anterior cervical and thoracic vertebral bodies, suggesting DISH. (**C**) Preoperative visual fluoroscopic swallowing study revealed moderate dysphagia in the oral and pharyngeal phases with incomplete closure of the epiglottis. (**D**) Postoperative laryngoscopy showed disappearance of the protruding mass at the posterior pharyngeal wall. (**E**) There was no evidence of regrowth of the osteophyte at 1-year postoperative radiograph.

A 65-year-old man visited the department of otolaryngology presenting with dysphagia and odynophagia for 3 months. Although he was able to have an oral diet, he felt severe difficulty in swallowing both solid and liquid food. He complained of a 12-kilogram reduction in body weight due to difficulty swallowing. He had undergone gastrectomy for stomach cancer 13 years ago and was taking psychiatric medications for bipolar disorder. Laryngoscopic examination revealed a protruding mass at the posterior pharyngeal wall obstructing the laryngeal entrance (Figure 2). Lateral cervical radiography and CT revealed a bridging osteophyte at the anterior cervical and thoracic vertebral bodies, suggesting DISH. Preoperative VFSS revealed moderate dysphagia in the oral and pharyngeal phases with incomplete closure of the epiglottis. For solid food, there were large amounts (>50%) of residue at the vallecular pouch and a moderate amount (10–50%) at the pyriform sinus. For liquid, direct aspiration to the trachea was identified. As conservative treatment with a dysphagia diet was not effective, he was referred to the spine center for surgical removal of the osteophyte. The surgery was performed via the modified Smith–Robinson approach and took 50 min with blood loss of less than 50 mL. Bone wax was applied to the bony margin after osteophyte removal. His symptoms markedly improved immediately after the surgery. There was no evidence of regrowth of the osteophyte at 1-year postoperative follow-up.

## 3. Discussion

**Figure 3:**
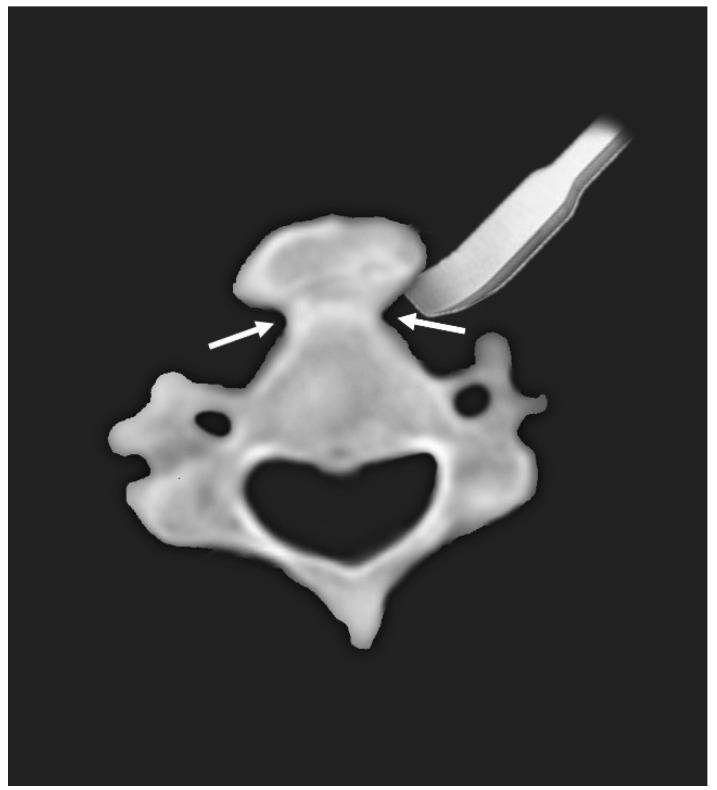
Diagram of typical axial appearance of anterior bony excrescence. Using the bottleneck point (arrows) between the vertebral body and bony excrescence, anterior osteophytes can be easily removed using an osteotome.

In the present study, we report two cases of dysphagia due to anterior osteophytes with underlying DISH. The patients were successfully treated with osteophyte removal via anterolateral approach. Based on axial images of preoperative CT, the osteophytes could be removed easily using an osteotome starting from the bottleneck point between the vertebral body and the excrescent osteophyte (Figure 3). This case report is a unique study focusing on the surgical aspect of treatment.

Any disruption in the swallowing process can be defined as dysphagia [13]. Dysphagia is a growing health concern in the aging population, and conservative estimates suggest that 15% of the elderly population is affected by it [9,14]. Conditions that may contribute to dysphagia include neurological disorders (i.e., stroke, dementia, traumatic brain injury, and Parkinson’s disease), rheumatoid disease (i.e., polydermatomyositis, progressive systemic sclerosis, and Sjögren’s disease), tumors involving the aerodigestive tract, chemotherapy, and radiotherapy [13]. According to previous literature, the management of dysphagia consists mainly of conservative measures, including compensatory management, provision of alternate nutrition such as non-oral feeding, and swallowing rehabilitation [9,13].

In contrast to dysphagic patients with other medical conditions, the role of conservative treatment seems limited in patients with DISH-related dysphagia. Conservative treatments, including medical treatment (e.g., nonsteroidal anti-inflammatory drugs, corticosteroids, antibiotics, and anti-reflux drugs), dietary measures (e.g., intake of fluid/soft foods and placement of gastrostomy), and pain relief (e.g., analgesics and cervical collar), have been proposed for patients with DISH-associated dysphagia [12,15,16,17]. However, the results of conservative treatment are inconclusive, and long-term outcomes of conservative treatment have not been reported [10]. Verlaan et al. reported in a systematic review that only 8 out of 31 patients reported an improvement of symptoms (7 patients did not improve and 16 cases were not specified). Moreover, although conservative methods may offer temporary relief in patients with dysphagia, long-term treatment is often difficult because of its poorly understood etiology [18].

Recent studies have reported excellent clinical outcomes following surgical removal of anterior osteophytes in patients with DISH-related dysphagia. Chung et al. reported that 16 of 21 patients showed an improvement in symptoms at one month postoperatively [11]. Mattiololi et al. also reported that all 21 patients demonstrated improvement to some degree following surgical removal of osteophytes [1]. According to a systematic review by Verlaan et al., most patients (91 of 92) showed an improvement in symptoms following surgery in cases where treatment outcomes were specified [4]. Moreover, if performed by experienced surgeons, surgery is low-risk and is well tolerated even in older patients [19]. Therefore, surgical management should be considered a principal option for patients with DISH-related dysphagia.

Osteophytectomy can be performed safely and quickly using the bottleneck point between the vertebral body and the bony excrescence. The surgery took less than an hour in both patients. Although the majority of studies did not report the detailed surgical technique for osteophyte removal, most of the available studies used high-speed drills for the removal of osteophytes [11,20,21]. However, when drilling within a large osteophyte, a surgeon can be confused regarding the extent and depth of bony removal, which can result in incomplete removal of the osteophyte. Furthermore, a high-speed drill can cause devastating injury to the visceral structure due to the sharp flutes of the burr. Although wide exposure with dissection of the neurovascular structure could decrease instrument-related injury, such a strategy can result in complications associated with wide exposure. In a study by Mattioli et al., more than half of the patients (12/21) underwent elective tracheotomy for wide prevascular retrovisceral exposure, and three of them experienced additional procedures due to postoperative complications (two postoperative hemorrhage and one postoperative dyspnea) [1]. As we could locate the bottleneck point between the vertebral body and the bony excrescence in all previous studies where CT images were available [1,10,17,18,20,22,23,24,25,26], we believe that this method can generally be applied to other cases.

Most previous studies reported on simple osteophytectomy via an anterolateral approach, while others have utilized cervical fusion with or without anterior plate fixation. The usefulness of cervical fusion has not yet been established; however, some advocate for performing cervical fusion to prevent late recurrence of osteophytes after surgery [27]. Although Miyamoto et al. reported recurrent osteophyte formation in all seven patients and revision surgery in two, Mattioli et al. reported that only 1 of 21 patients experienced asymptomatic recurrence at 9 years after surgery [1,27]. Anterior plate fixation, some argue, may cause additional esophageal compression, while others claim that the plate itself is not relevant to swallowing difficulty and could act as a physical barrier and suppress bony overgrowth [28,29]. Chung et al. reported that simple osteophytectomy alone tended to yield more favorable results than additional plate fixation on the Dysphagia Outcome and Severity Scale [11]. Considering that the revision rates due to recurrent osteophytes are not high and patients are generally old, it would be reasonable to perform simple osteophytectomy with long-term surveillance in patients with DISH-related dysphagia. However, the short-term period of follow-up was not enough to assess the recurrent osteophyte and thus a major weakness in this study. Therefore, a longer term of follow-up is necessary.

## 4. Conclusions

In the present study, we report two cases of DISH-related dysphagia. The anterior osteophyte was removed using an osteotome, starting from the natural bottleneck point between the vertebral body and the bony excrescence. Patients’ symptoms improved following osteophyte removal, without recurrence. In cases of DISH-related dysphagia, osteophyte removal using an osteotome could improve dysphagia safely and quickly.

## Data Availability

Not applicable.
